# 

**Published:** 1882-06

**Authors:** 


### CONTENTS.

| | |
|---|---|
| Care of Infants | 283 |
| Croup | 284 |
| Moles, Warts and Birth Marks | 285 |
| Mother and Infant | 286 |
| Can Animals Talk | .28 |
| The Coming Man Physically | 290 |
| The Pronunciation of "U." | 291 |
| Removal of Stains and Spots | 292 |
| Goethe and Schiller | 294 |
| Life and Death | 297 |
| Health Maxims | 297 |
| A Happy Home | 304 |
| Lead Colic | 306 |
| The Teeth | 307 |
| Sea Sickness | 308 |
| The Emotions | 318 |
| Appetite | 319 |
| Decision of Character | 320 |
| Medical Progress | 322 |
| Be Systematic | 323 |
| Earliest Signs of Consumption | 324 |
| Coughing | 326 |
| Health | 327 |
| Habit | 328 |
| Out-Door Safety | 329 |
| Sprains | 330 |
| Heart Affections | 331 |
| Small Pox | 332 |
| Ice Water | 333 |
| Regimen | 334 |
| Sleeping | 335 |
| Tobacco Users | 337 |
| Climate for Consumptives | 338 |
| Animal Ailments | 339 |

